# Human immunodeficiency virus infection and cerebral malaria in children in Uganda: a case-control study

**DOI:** 10.1186/1471-2431-11-5

**Published:** 2011-01-14

**Authors:** Peace D Imani, Philippa Musoke, Justus Byarugaba, James K Tumwine

**Affiliations:** 1 Department of Paediatrics and Child Health, School of Medicine, College of Health Sciences, Makerere University, P.O Box 7072, Kampala Uganda; 2 Makerere University-Johns Hopkins (MU-JHU) Research Collaboration, Upper Mulago Hill Road, P.O. Box 23491, Kampala - Uganda

## Abstract

**Background:**

Human immunodeficiency virus (HIV)-1 infection increases the burden of malaria by increasing susceptibility to infection and decreasing the response to malarial treatment. HIV-1 has also been found to suppress the immune system and predispose to severe forms of malaria in adults. There is still a paucity of data on the association between HIV-1 infection and cerebral malaria in children. The aim of this study was to determine whether HIV-1 infection is a risk factor for cerebral malaria in children.

**Method:**

We conducted an unmatched case-control study, in which 100 children with cerebral malaria were compared with 132 with uncomplicated malaria and 120 with no malaria. In stratified analyses we estimated odds ratios (ORs) and 95% confidence intervals (CIs) adjusted for age.

**Results:**

HIV-1 infection was present in 9% of children with cerebral malaria compared to 2.3% in uncomplicated malaria (age-adjusted odds ratio (aOR) 5.94 (95% confidence interval (CI) 1.36-25.94, p = 0.012); and 2.5% in children with no malaria (aOR 3.85 (95% CI0.99-14.93, p = 0.037). The age-adjusted odds of being HIV-positive among children with cerebral malaria compared to the control groups (children with uncomplicated malaria and no malaria) was 4.98 (95% CI 1.54-16.07), p-value = 0.003.

**Conclusions:**

HIV-1 infection is associated with clinical presentation of cerebral malaria in children. Clinicians should ensure that children diagnosed with HIV infection are initiated on cotrimoxazole prophylaxis as soon as the diagnosis is made and caretakers counselled on the importance of adherence to the cotrimoxazole towards reducing the risk of acquiring *P.falciparum *malaria and associated complications such as cerebral malaria. Other malaria preventive measures such as use of insecticide-treated mosquito nets should also be emphasized during counselling sessions.

## Background

Malaria and Human immunodeficiency virus (HIV)-1 are two of the most common global health challenges today and the two infections commonly overlap in distribution in most countries especially in sub-Saharan Africa[1]. Studies have demonstrated interaction between these two infections with the majority of studies conducted in adults [2-6]. HIV-1 infection has been found to be associated with severe forms of malaria and particularly cerebral malaria in adults but there is still a paucity of information on the interaction between the two infections in children [2,7,8].

HIV-1 infected adults have a greater percentage of severe malaria episodes with more frequent hospitalizations compared to their uninfected counterparts and are at a greater risk of cerebral malaria [2,3,7,9]. Infection with HIV-1 leads to impaired immune response to malaria through cellular immunosuppression resulting in a higher likelihood of increased parasitaemia and severe malarial infections. Multiple studies have indicated an alteration in cytokine levels in those co-infected with malaria and HIV [10-12].

Malaria-specific antibodies, the main target of protective malaria immunity seem to be little impaired in early HIV infection but in advanced AIDS, evidence indicates B-cell stimulation is diminished to some extent, resulting in decreased production of malaria antibodies [10,13]. HIV infection has also been found to increase the susceptibility to new malarial infections [3,9]. There is evidence also showing strong correlation between decreasing CD4+ cell counts and both the levels of parasitaemia and the patients' clinical status [12,14]. HIV- infected children with advanced immunosuppression have more episodes of clinical malaria and higher parasite densities compared to those children without advanced immunosuppression [15]. In children, cerebral malaria is the most severe neurological complication of *P. falciparum *malaria infection and has a case fatality ranging from 5 to 40% [16-19].

Whether HIV-1 infection increases the risk of cerebral malaria in children is not known. We conducted this study to determine whether HIV infection is a risk factor for cerebral malaria in children from Uganda.

## Methods

We conducted a case-control study of children with cerebral malaria recruited from Mulago hospital Paediatric emergency unit. We also recruited 132 children with uncomplicated malaria and 120 with no malaria as controls from the paediatric out patients units. Cases included children aged below 12 years with cerebral malaria [defined as a clinical syndrome characterized by coma: Blantyre Coma Scale(BCS) ≤ 2] for more than 30 minutes after termination of a seizure or correction of hypoglycaemia, detection of asexual forms of *P falciparum *malaria parasites on peripheral blood smears, and a normal cerebrospinal fluid (CSF)[20]. The parasite density was determined after enrolment and was therefore not considered in the inclusion criteria. Severe anemia was not an inclusion criterion per se but children with severe anemia, who had a history of convulsions and were admitted with a BCS ≤ 2 (in coma), with a relatively normal blood sugar (at least above 2.2 mmol/l), normal electrolytes and a normal CSF with a positive blood smear for malaria parasites were taken as having cerebral malaria. The control group with uncomplicated malaria included children aged below 12 years, with fever and a positive malarial smear. Uncomplicated malaria was defined as fever with a positive blood smear for malaria parasites but no features of severe malaria which include convulsions, inability to drink, vomiting, and drowsiness, and lethargy, loss of consciousness, respiratory distress, or severe anaemia. Axillary temperature was measured and fever was considered above 37.6°C. A temperature above 39.5°C was considered hyperpyrexia. For the control group with no malaria, we included children aged below 12 years, with no symptoms of malaria such as fever, malaise or weakness and a negative blood smear for malaria parasites. Children who had asymptomatic parasitaemia (positive blood smear with no fever) were excluded from the control group these constituted 10% of 133 children screened with no malaria.

All children recruited in this study were of unknown HIV status at time of enrolment and therefore were not on cotrimoxazole prophylaxis. We required 100 cerebral malaria cases to determine the association between HIV infection and cerebral malaria, in a case to control ratio of 1:1, with a power of 80% and 95% confidence interval according to Fleiss formula for case control studies [21]. We continued to enrol controls until all the required cases of cerebral malaria were recruited

A questionnaire was used to collect information such as the socioeconomic, demographic characteristics, clinical history, and physical examination as well as laboratory findings. We consecutively enrolled patients who fulfilled the inclusion criteria for the three study groups until the sample size was achieved. Cases and controls were recruited at the same time. Patients were examined by a qualified medical officer who documented all the findings. A trained counsellor was available to do both pre- and post-test counselling for HIV. Five millilitres of blood were drawn from each child with cerebral malaria and 2-3 millilitres in children with uncomplicated malaria for a malaria smear, *P*. *falciparum *parasite density, HIV serology and CD4 cell count, blood glucose and serum electrolytes which were done in different laboratories. A finger prick was done for children with no malaria for the blood smear and rapid HIV test and a blood sample for CD4 count taken if the child was found to be HIV positive. The HIV tests were rapid and the children who were HIV positive had blood sent for CD4 cell count immediately. These were used to stage level of immunosuppression according to the WHO guidelines[22]. No repeat CD4 test was carried out after treatment. Serum electrolytes were assessed on all children with cerebral malaria. A lumbar puncture was performed on the children with suspected cerebral malaria and a normal CSF described as a specimen with a cell count of ≤5 WBCs/mm^3 ^and a CSF protein of ≤40 mg/dl. Patients with cerebral malaria and those with uncomplicated malaria received standard care according to the Mulago Hospital Department of Paediatrics guidelines. Those without malaria were seen by the surgeons who offered appropriate treatment for the surgical condition. HIV positive children were referred to the Paediatric Infectious Disease Clinic (PIDC) where they were evaluated, managed and followed up. The number of asexual parasites per 200 white blood cells (WBCs) was counted and parasite densities computed by assuming a mean WBC count of 8,000/litre (WHO)[23].

The primary objective of this study was to assess HIV-1 as an independent risk factor for cerebral malaria. The secondary objective was to determine the prevalence of HIV-1 infection in children with cerebral malaria and the correlation between the level of immunosuppression (CD4+ T-cell count) and parasite density.

## Statistical Analysis

Data was entered into EPI-DATA 3.1 software package and analysed using the statistical program SPSS version 13 software packages. Categorical variables were summarized as proportions while means and standard deviation were used for normally distributed continuous variables. Medians with corresponding inter-quartile ranges were used for skewed data. The Chi- square test was used to compare categorical variables between those cerebral malaria and those without. Multivariate analysis (logistic regression by backward method) was then used to tease out whether HIV infection is independently associated with clinical cerebral malaria.

## Ethics and Consent

Informed consent to participate in the study was obtained from the caretakers after explanation of the study objectives and relevance, risks and benefits. A separate consent for HIV testing was also obtained. Laboratory results were available for patient management. Approval was obtained from the Makerere University Faculty of Medicine, Research and Ethics Committees, Mulago Hospital, and the Uganda National Council of Science and Technology.

## Results

**Figure 1:**
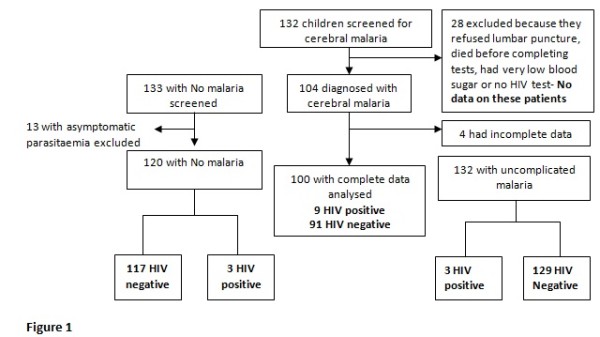
**Study profile**.

The study was conducted from November 2006 to March 2007 during which 352 children were recruited. One hundred had cerebral malaria (CM), 132 had uncomplicated malaria (UM) and 120 had no malaria (NM). Thirty two patients with suspected cerebral malaria were excluded, 28 of whom we could not do complete assessment and 4 had incomplete data. Ten percent (13/133) of children with no malaria were found with asymptomatic parasitaemia and these were also excluded from the analysis Figure 1. Children hospitalized with cerebral malaria were more likely to be less than five years of age (81% vs 68% and 63%) and not to use mosquito nets (54% vs 86% and 63% in the uncomplicated malaria and no malaria arms respectively). Of the 352 children enrolled, 3 (2 cases and 1 control) had moderate to severe wasting (Table 1a).

**Table 1:** Baseline characteristics of study population

a. Characteristics of all children and their	Cerebral malaria	Uncomplicated malaria	No malaria
**care takers**	**n = 100**	**n = 132**	**n = 120**

**Variable**	**N (%)**	**N (%)**	**N (%)**

**Child factors**			
**Age (Months)**			
≤59	81 (81)	90 (68.2)	76 (63.3)
>59	19 (19)	42 (31.8)	44 (36.7)
**Sex M:F**	01:00.7	01:00.7	01:00.7
**Median duration of fever in days (IQR)**	3 (2-4)	2 (1-4)	-
**Use of mosquito net**			
Yes	54 (54)	86 (65.2)	63 (52.5)
No	46 (46)	46 (34.8)	57 (47.5)
**Immunization status**			
Fully/up-to-date	73 (73)	102 (77.3)	105 (87.5)
Partial/uncertain	27 (27)	30 (22.7)	15 (12.5)
**Pre-hospital treatment**			
Yes	82 (82)	76 (57.6)	-
No	18 (18)	56 (42.4)	
**Median parasite density (IQR)**	23480 (1660-145960)	21680 (7350-54300)	
**HIV status**			
Positive	9 (9)	3 (2.3)	3 (2.5)
Negative	91 (91)	129 (97.7)	117 (97.5)
**Caretaker factors**			
**Age of caretaker (in years)**			
≤30	70 (70.0)	103 (78.0)	63 (52.5)
>30	30 (30.0)	29 (22.0)	57 (47.5)
**Antimalarial medicines kept at home**			
Yes	28 (28)	36 (27.3)	25 (20.8)
No	72 (72)	96 (72.7)	95 (79.2)
**Caretaker's preferred first option**			
H/U	25 (25)	44 (33.3)	93 (77.5)
Other^2^	75 (75)	88 (66.7)	27 (22.5)
**Number of children <5y in the household**			
≤3	83 (83)	124 (93.9)	79 (84.9)
>3	17 (17)	8 (6.1)	14 (15.1)

**b. Characteristics of cerebral malaria patients by HIV status**	**HIV+ (n = 9)**	**HIV- (n = 91)**	**P-value**
	**n (%)**	**n = (%)**	**OR (95% CI)**

**Age**			
≤59 Mo	7 (77.8)	74 (81.3)	
>59 Mo	2 (22.2)	17 (18.7)	
**Duration of fever (days)**			
≤3	5 (55.6)	59 (35.2)	0.58
>3†	4 (44.4)	32 (64.8)	1.48 (0.27-7.37)
**Median number of convulsions (IQR)**	3 (2-3)	2 (1-5)	
**Pre-hospital treatment**			
Yes†	6 (66.7)	76 (83.5)	<0.001
No	3 (33.3)	15 (16.5)	3.36 (1.81-6.21)
**Median parasite density (IQR)**	240,000 (3080-583520)	21,440 (1600-133080)	
**Parasite density/μl**			
>500,000†	2 (22.2)	2 (2.19)	0.003
≤500,000	7 (77.8)	89 (97.8)	12.71 (1.56-104.41)

n = number of children, IQR = Inter-quartile range, ^1 ^skilled laborers, ^2 ^tepid sponging/self-medication/herbs, H/U = health unit, † reference group

Children with cerebral malaria also had median serum sodium of 137.0 mmol/L (range 131-142 mmol/L). One patient had severe hypoglycaemia at the time of enrolment (blood sugar below 2.2 mmol/L). The median blood sugar was 7.4 mmol/L (range 5.9-9.5 mmol/L). Thirty eight percent of patients with CM had severe anaemia (Hb < 5 g/dl). No microbiological culture or virological tests were done on the CSF since the white blood cell count was not raised.

Children with cerebral malaria who were also HIV positive children were more likely to have had fever for more than three days, been on treatment prior to this admission, present with higher temperatures (>38°C) and have a higher parasite density (Table 1b). HIV positive children with cerebral malaria were also more likely to have a higher parasite density. The unadjusted odds of having parasite density greater than 500,000/μl with cerebral malaria was 12.71 times higher in HIV positive children (95% CI; 0.77-189.57) compared to those who were HIV negative. The age-adjusted OR was 13.76 (95% CI; 1.24-153.11), p-value = 0.0051.

Sixty seven (56%) of the 120 controls with no malaria recruited from SOPD had inguino-scrotal hernias, 26 (22%) presented with hydroceles, and the others had congenital anorectal malformations, umbilical hernias, undescended testis, rectal prolapse and different types of cysts. None of these controls required urgent intervention but only an appointment for elective surgery. Ideally, these controls (no malaria) would have been selected from similar communities as the cases but this was not the case due to limited funds for this. This could have potentially caused bias in the HIV prevalence. The prevalence of HIV was 9% among children with cerebral malaria compared to 2.3% and 2.5% in the uncomplicated malaria and no malaria groups respectively.

There was an association between HIV infection and clinical cerebral malaria, HIV infected children were more likely to have cerebral malaria than the HIV negative ones, unadjusted OR = 4.05 (95%CI 1.24-14.19), p = 0.006 and age-adjusted odds ratio (aOR) was 4.98 (95%CI 1.54-16.07) p = 0.003. Among children below the age of 5 years, the HIV prevalence in cerebral malaria cases was 8.6% (7 of 81) compared to 1.2% (2 out of 166) among all controls. The odds of presenting with clinical cerebral malaria below the age of five was higher among children who were HIV positive compared to the HIV negative counter parts OR = 7.76 (95% CI; 1.42-77.6); p-value 0.003 (Table 2).

**Table 2:** Comparing cerebral malaria and no malaria groups

Variable	Cerebral Malaria	No Malaria	Unadjusted	p-value
	n = 100	n = 120	p-value	Adjusted OR (95%CI)
	**N (%)**	**N (%)**	**OR (95%CI)**	

**Age (Months)**				
≤59†	81 (81)	76 (63.3)	0.004	0.004
>59	19 (19)	44 (36.7)	2.47 (1.27-4.88)	2.49 (1.30-4.75)¶
**Use of mosquito net**	54 (54)	63 (52.5)	0.825	0.968
Yes†	46 (46)	57 (47.5)	1.06 (0.62-1.81)	0.99 (0.57-1.71)‡
No				
**HIV status**	9 (9)	3 (2.5)	0.035	0.037
Positive†	91 (91)	117 (97.5)	3.86 (0.99-14.91)	3.85 (0.98-15.07)‡
Negative				
**Caretaker's preferred first option H/U**				
Yes	25 (25)	93 (77.5)	<0.001	<0.001
No†	75 (75)	27 (22.5)	10.33 (4.96-21.52)	9.93 (4.77-20.68)‡
**Number of children <5y/household**	83 (83)	79 (84.9)	0.714	
≤3	17 (17)	14 (15.1)	1.16 (0.53-2.51)	
>3†				

n = number of children, CI confidence interval, OR odds ratio, H/U = health facility, † reference group, ¶ adjusted for HIV, ‡adjusted for age, CM = cerebral malaria, NM = no malaria

On comparing the children with cerebral malaria to those with the no malaria, the age-adjusted odds of presenting with cerebral malaria was 3.85 times higher among HIV positive children compared to HIV negative ones, (95% CI = 0.99-14.93) p = 0.037. The aOR was 5.94 (95% CI 1.36-25.94) when comparing children with cerebral malaria to children with uncomplicated malaria, p-value 0.012 (Table 3). Table 4 shows a summary of the age-adjusted odds ratios comparing the cases to the control groups

**Table 3:** Comparing cerebral malaria and uncomplicated malaria

	Cerebral Malaria	Uncomplicated Malaria	Unadjusted	
**Variable**	**n = 100**	**n = 132**	**p-value**	**p-value**
	**N (%)**	**N (%)**	**OR (95%CI)**	**adjusted OR (95% CI)**

**Age (Months)**				
≤59†	81 (81)	90 (68.2)	0.028	0.016
>59	19 (19)	42 (31.8)	1.99 (1.07-3.69)	2.41 (1.14-4.02)‡
**Use of mosquito net**	54 (54)	86 (65.2)	0.086	0.051
Yes†	46 (46)	46 (34.8)	0.63 (0.37-1.07)	0.58 (0.34-1.01)¶
No				
**Pre-hospital treatment**	82 (82)	76 (57.6)	<0.001	0.002
Yes†	18 (18)	56 (42.4)	3.36 (1.81-6.21)	3.02 (1.52-5.99)¶
No				
**Median parasite density/μl**	4 (4)			
>500,000†	96 (96)	1 (0.76)	0.092	0.069
≤500,000		131 (99.24)	5.46 (0.53-270.77)	5.23 (0.71-38.59)¶
**HIV status**				
Positive†	9 (9)	3 (2.3)	0.022	0.012
Negative	91 (91)	129 (97.7)	4.25 (1.10-16.44)	5.94 (1.23-28.72) ¶
**Number of children <5y/household**				
≤3	83 (83)	124 (93.9)	0.008	0.005
>3†	17 (17)	8 (6.1)	3.18 (1.31-7.69)	4.03 (1.51-10.76)¶

CI = confidence interval, OR odds ratio, ‡ adjusted for HIV status, ¶ age-adjusted OR, † reference group, CM = cerebral malaria, UM = uncomplicated malaria

**Table 4:** Association between cerebral malaria and HIV infection comparing CM to both control groups

	CM and UM	CM and NM	CM and (NM + UM)
	**OR (95% CI)**	**OR (95% CI)**	**OR (95% CI)**

**All children**	5.94 (1.23-28.72)**‡**	3.85(0.98-15.07) **‡**	4.98 (1.54-16.07)**‡**
**Excluding children >5 year**		3.50 (0.69-17.34)	7.76 (1.52-39.47)

‡ adjusted for age, CM = cerebral malaria, UM = uncomplicated malaria, NM = no malaria

The CD4 lymphocyte percentage and absolute counts were used to determine the stage of HIV immunosuppression. Twenty percent HIV-positive children had their stage of immune suppression by CD4 count corresponding to their WHO clinical stage. Fifty five percent of the HIV-infected children with cerebral malaria were not clinically immunosuppressed or just had mild immunosuppression compared to 16.7% of HIV positive children with uncomplicated or no malaria. Children with cerebral malaria were more likely to have mild to moderate immunosuppression than were children without cerebral malaria (age-adjusted OR = 3.33 (95%CI 0.25-45.11); p-value = 0.336). However, the numbers of HIV positive children in this sample was very small.

## Discussion

Our results are in agreement with studies conducted in Kwazulu Natal, South Africa and in Kenya where researchers in those countries also found similar association with HIV infection and severe malaria in children [9,24,25]. In South Africa, the prevalence of HIV among children with severe malaria was 17% compared to 7.5% in children with uncomplicated malaria while the study in Kenya, found a prevalence of 12% among children admitted with severe malaria. In both studies, the forms of severe malaria which the children presented with and HIV prevalence in cerebral malaria were not reported.

The prevalence of HIV was lower than expected in this study population probably because of early HIV testing and diagnosis and initiation of cotrimoxazole prophylaxis for children found to be HIV positive which would also be protective against *P. falciparum *malaria and severe forms of malaria [14,26,27]. None of the HIV positive patients had been on prior cotrimoxazole prophylaxis since their HIV status was unknown at the time of enrolment.

HIV infected children in this study were more likely to have hyperparasitaemia (parasite density >500,000/μl) which was consistent with what was found in studies in the Kenya y and Zimbabwe studies in which HIV infection was associated with higher parasite density [2,25]. Presumably, HIV infected children are less likely to clear parasites from their blood than the non HIV infected children because HIV infection impairs the immune response to malaria parasites, leading to decreased ability to control parasitaemia [2,5,28,29]. The high parasitaemia places these children at a higher risk of developing cerebral malaria because of the increased likelihood of having the parasites sequester in the capillary blood vessels. It is also possible that HIV-infected children are at increased risk of developing cerebral malaria because of an increased production of nitric oxide synthase, ICAM-1 and VCAM-1 with HIV infection [30]. Nitric oxide has been suggested as a cause for coma because of its ability to interfere with neurotransmission in the brain [30,31]. However, other studies have found no significant association with cerebral malaria [32].

Due to the low number of HIV infected children in our controls, we found no association between cerebral malaria and low CD4 cell count. A longitudinal study in Malawi has already indicated that lower CD4 cell counts were associated with higher incidences of pneumonia, sepsis, and tuberculosis but not of malaria([33].

Other factors found to be associated with an increased risk of presenting with clinical cerebral malaria were age below five years and not using a mosquito net. These findings are also consistent with previous studies [16,34,35].

Our findings support the hypothesis that HIV infection is a risk factor for cerebral malaria in children. The findings from this study add to the existing knowledge of interaction between HIV infection and *P. falciparum *malaria in children and are of both clinical and public health importance.

## Conclusions

HIV-1 infection is associated with clinical presentation of cerebral malaria in children. Clinicians should ensure that children diagnosed with HIV infection are initiated on cotrimoxazole prophylaxis as soon as the diagnosis is made and caretakers counselled on the importance of adherence to the cotrimoxazole towards reducing the risk of acquiring *P.falciparum *malaria and associated complications such as cerebral malaria. Other malaria preventive measures such as use of insecticide-treated mosquito nets should also be emphasized during counselling sessions.

## Study Limitations

There are sources of potential sources of bias that cannot be eliminated in this study. These inherent sources include selection of no malaria controls from a hospital setting which may have affected our results. We however tried to limit this by selecting hospital controls from similar communities. We also excluded children with conditions clearly documented to be associated with HIV such as pneumonia, severe malnutrition, persistent diarrhoea, and chronic otitis media. The design of the study may have also affected the results. The case control design may have led to exaggeration of the risk because cases and controls were not matched on characteristics such as age, maternal characteristics and others. The parasite density may not be a true reflection of the level of parasitaemia since the individual WBC count was not available to estimate the parasite density but instead assumed 8,000/μl. There is a possibility that the WBC count was different in HIV+ and HIV- children.

Children with cerebral malaria were not followed up to determine whether the outcome depended on HIV serostatus. The strength of the study is that there were more than one control groups and given that cerebral malaria is a relatively rare complication of malaria, it was an appropriate study design to conduct given the time.

## Potential conflicts of interest

The authors declare that they have no competing interests.

## Authors' contributions

PDI conceived and designed the study, performed analysis and interpretation of data and drafted the manuscript. PM, JB and JKT assisted with the design, interpretation of data and the critical review of the manuscript. All authors read and approved the final manuscript and participated in its revision. JKT is the guarantor of the work.

## Pre-publication history

The pre-publication history for this paper can be accessed here:

http://www.biomedcentral.com/1471-2431/11/5/prepub
